# Immunotherapy in ovarian cancer: spatial functional genomics to unravel resistance mechanisms

**DOI:** 10.1038/s41392-024-02110-w

**Published:** 2025-01-22

**Authors:** Martina Rausch, Karlotta Bartels, Josef Leibold

**Affiliations:** 1 Cluster of Excellence iFIT (EXC2180) “Image-guided and Functionally Instructed Tumor Therapies” University of Tübingen, Tübingen, Germany; 2 Department of Medical Oncology and Pneumology, University Hospital Tübingen, Tübingen, Germany; 3 Department of Pediatric Hematology and Oncology, University Children’s Hospital, University of Tübingen, Tübingen, Germany

**Keywords:** Tumour heterogeneity, Cancer microenvironment, Tumour immunology, Gynaecological cancer

The recent publication by Mollaoglu et al.^1^ in *Cell* reveals an unexpected role for tumor derived IL4 in driving immunotherapy resistance in ovarian cancer (OvCa). This finding nominates the combination of immunotherapy and IL4-signaling targeting strategies as a promising new approach for the treatment of advanced OvCa.

Ovarian Cancer (OvCa) is the third most common gynecological malignant disease affecting women.^2^ It is often diagnosed at late stages and is characterized by heterogenous features with limited treatment options. Initial response to standard of care platinum-based chemotherapy combined with surgery is often followed by disease relapse and subsequent death of patients. Despite the recent success of immune checkpoint inhibition in different cancer entities, most OvCa patients do not benefit from immunotherapy-based treatment approaches. The responsiveness of ovarian tumors to immune checkpoint blockade (ICB) is thereby hindered by weak immunogenicity due to low mutational burden and an immune suppressive tumor microenvironment (TME) characterized by heterogenous immune cell infiltration.^3^ Still, functional evidence for key factors that govern cancer cell-immune cell interaction and drive immunotherapy resistance in OvCa remains limited.

In their study, Mollaoglu et al. highlight how OvCas shape the TME through extracellular factors, transforming it into a tumor promoting organ. With a focus on cancer cell-macrophage interactions, the study exemplifies how a better understanding of these dynamics could result in improved outcome of immunotherapeutic approaches.

**Fig. 1:**
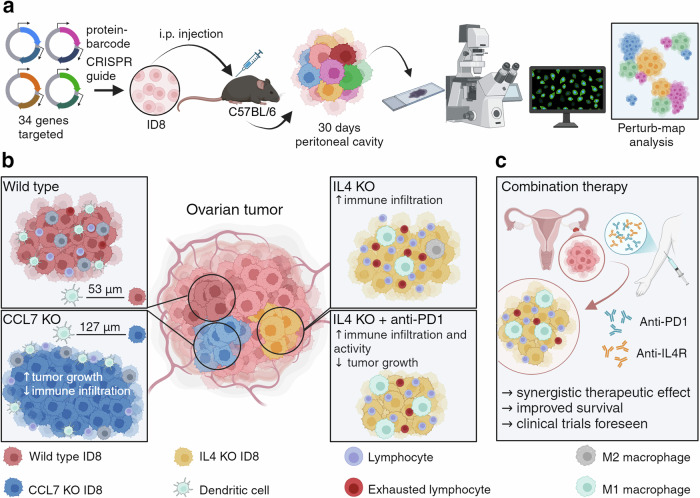
A spatial functional genomics screening approach identified tumor-derived IL4 to orchestrate resistance to immune checkpoint inhibition in ovarian cancer (OvCA). **a** Performing a Perturb-map CRISPR screen Mollaoglu et al.^1^ assessed how 34 genes relevant for cancer cell-macrophage interaction impact tumor growth and immunotherapy response in a transplantation based OvCa mouse model (ID8 cells). **b** CCL7 knockout (KO) in the tumor cells increased tumor growth and distance between cancer and immune cells generating an immune excluded tumor microenvironment (left). IL4 KO in the tumor cells resulted in an increased infiltration of functionally impaired T cells and a decrease of immunosuppressive M2 macrophages (upper right). The combination of IL4 KO and immune checkpoint blockade resulted in increased immune cell infiltration and a T cell mediated anti-tumor activity (lower right). **c** The combination of immune checkpoint blockade (anti-PD-1 targeting antibody) with FDA approved anti-IL4R targeting antibody dupilumab is a promising therapeutic strategy to treat OvCa patients. Created with BioRender.com

A key challenge in implementing ICB in OvCa patients is the intrinsic heterogeneity of the disease, encompassing diverse immunological, genetic and molecular aspects. Most preclinical OvCa tumor models only partially capture this heterogeneity, complicating efforts to investigate its underlying mechanisms and identifying novel therapeutic concepts. Assessing the functional consequences of secreted factors is further confounded by the need for spatial resolution, adding another layer of complexity to the analysis. Mollaoglu et al. overcome these investigational hurdles by leveraging a protein-barcode CRISPR (PC/CRISPR) library combined with Perturb-map^4^ (Fig. 1a), to visualize growth patterns of distinct cancer cell clones, their positioning within the tumor tissue, and their relation to infiltrating immune cells. This platform was integrated with an OvCa mouse model that closely replicates the disseminated and heterogeneous characteristic observed in human ovarian cancer.

The authors functionally assessed the role of *n* = 34 genes involved in cancer cell-macrophage interaction in OvCa. They identified loss of *Plaur*, *Mif* and *Serpine 1* to be responsible for a growth disadvantage of murine ID8 OvCa cells in vivo, whereas *Ccl3*, *Calr* and *Ccl7* knockout (KO) tumor cells were substantially enriched and formed distinct lesions within the tumors. Mechanistically, CCL7 KO resulted in a reduction of immune cell infiltration and an increased distance between cancer and immune cells in the TME (Fig. 1b). The authors further correlated CCL7 expression with immune cell infiltration in a TCGA dataset of human OvCa, suggesting that CCL7 contributes to an immune-excluded TME, potentially impeding immunotherapy-based treatment approaches. Remarkably, while CCL7 is a secreted factor, the observed effects were not compensated by surrounding chemokine secreting clones, highlighting the impact of soluble factors in shaping heterogeneity within distinct niches.

To further elucidate mechanisms of immunotherapy resistance, the researchers repeated the Perturb-map analysis using the same PC/CRISPR-edited murine OvCa tumors in the context of anti-PD-1 blockade. The authors identified IL4 KO tumor cells among the genetic clones with a significant reduction in relative abundance only in context of immune checkpoint blockade, suggesting that IL-4 promotes resistance to anti-PD-1 treatment. Mollaoglu et al. demonstrated that OvCa cells are a relevant source of IL-4, which is expressed under the transcriptional control of GATA6 in the tumor cells. Tumor cell derived IL-4 fostered an immunosuppressive TME, characterized by tumor-associated M2 macrophages and limited T cell infiltration. Notably, combining anti-PD-1 therapy with either a genetic IL-4 KO in tumor cells or with an FDA-approved IL4R-blocking antibody in a clinically relevant OvCa mouse model delayed tumor progression underscoring the translational potential of this strategy as a therapeutic approach (Fig. 1c). Importantly, clinical trials applying this strategy in patients with non-small cell lung cancer (NSCLC) are already underway and its success remains to be determined^5^ (NCT05013450).

Mollaoglu et al. elegantly used a spatial functional genomics approach to mechanistically unravel how tumor derived secreted or non-secreted factors impact tumor cell-immune cell interactions and ultimately immunotherapy response. They identified several distinct factors from the cancer cell-macrophage axis impacting tumor growth and tumor microenvironment composition and thereby determining responsiveness to ICB.

Of note, the selective pressure sustained by anti-PD-1 immune checkpoint blockade revealed previously concealed candidates and also uncovered ambiguous functions of distinct chemokines such as CCL3. The authors initially identified CCL3 as a factor suppressing tumor growth in vivo; however, in contrast to CCL7, their screening approach under anti-PD-1 therapy suggests that it may also play a role in driving immune cell exclusion from the tumor site. This intriguing duality of CCL3 was not followed up by the authors and the underlying biology remains elusive.

Mollaoglu et al. build their study on perturbing genes predicted to be relevant for tumor cell-macrophage interactions. Using a similar approach, future studies should additionally delineate the interplay between tumor cells and other crucial cell types in the OvCa TME such as e.g., T cells, B cells or neutrophils, (cancer associated) fibroblasts, and endothelial cells. Furthermore, the authors identified GATA6 to regulate IL-4 production in OvCa cells. Elucidating the upstream determinants of GATA6 activation and linking the secretory phenotype of the tumor cells to the underlying genetic landscape could result in further insights into TME immunological niches and ultimately aid in stratifying patients who are most likely to benefit from such novel therapeutic approaches.

In conclusion, the study by Mollaoglu et al. underlines that distinct immunological neighborhoods in heterogenous solid tumors determine the outcome of immunotherapies. In addition, the study highlights the potential of combinatorial treatment approaches that take tumor characteristics and microenvironmental factors into account and might thus result in synergistic activity and improved clinical outcomes.
